# Identification of Streptomyces strains isolated from Humulus lupulus rhizosphere and determination of plant growth promotion potential of selected strains

**DOI:** 10.3906/biy-1906-37

**Published:** 2019-12-13

**Authors:** Fadime ÖZDEMİR KOÇAK

**Affiliations:** 1 Department of Nursing, School of Health, Bilecik Şeyh Edebali University, Bilecik Turkey; 2 Biotechnology Application and Research Center, Bilecik Şeyh Edebali University, Bilecik Turkey

**Keywords:** Fourier transform infrared, plant-growth promoting rhizobacteria, Solanum lycopersicum, Streptomyces, 16S rRNA

## Abstract

In the present study it was aimed to obtain novel strains of actinomycetes that have the ability to promote plant growth. For this, seven soil samples from the rhizosphere of *Humulus lupulus* (Pazaryeri, Bilecik) were used and potential isolates were obtained. 16S rRNA genes of 30 isolates were amplified by PCR and sequenced. Eighteen isolates were found to be closely related to *Streptomycetes *spp. and they were tested for their proteolytic activity, cellulase activity, phosphate solubility, IAA production, biofilm formation, and growth in nitrogen-limited medium. Two isolates, HCI 12 and HCI 36, were selected according to the results of these tests and their effects on growth of tomato plants (*Solanum lycopersicum*) were determined. Results indicated that the HCI 12 and HCI 36 strains caused a significant increase in root and shoot lengths, dry/fresh weights, and vigor index values compared to the control. The change in macromolecular structure including carbohydrates, proteins, and lipids of tomato plants with HCI 12 and HCI 36 inoculation was determined by Fourier transform infrared (FTIR) analysis. The results obtained from FTIR analysis were used in principal component analysis to evaluate changes in amide, carbohydrate, and lipid ratios of plant leaves due to microorganism application. Inoculation with the HCI 12 and HCI 36 strains caused a significant increase in the total carbohydrate and lipid ratio of tomato.

## 1. Introduction

Actinomycetes are widely dispersed in habitats of animals, leaves, shoots, or rhizospheres of plants. Members of the actinomycetes have the ability to produce various bioactive secondary metabolites such as antibiotics, antitumor agents, immunosuppressant agents, and enzymes (Valli et al., 2012). Species like *Streptomyces*,* Micromonospora*,* Nonomuraea*, and* Kribbella* are known as bioactive metabolite producers. 

The genus *Streptomyces* can be widely found in rhizosphere soil and *Streptomycetes* species producing secondary metabolites were determined to have symbiotic relationships with plants, fungi, and animals rather than existing freely in soil. Certain species of insects, plants, and sea animals can protect themselves against infections thanks to this symbiotic relation (Seipke et al., 2011). Members of this genus are known to enhance plant growth (Sousa et al., 2008; Sathya et al., 2017). These bacteria can be effective in promoting plant growth directly or indirectly. Plant growth-promoting rhizobacteria (PGPR) used as a biofertilizer can produce indole acetic acid (IAA), a phytohormone that promotes the growth of plants, and fixate nitrogen from the atmosphere to be used by the plant, solubilize phosphate, and increase the level of phosphate imported by the plant and bind iron for the use of the plant. Endophytic *Streptomyces* isolated from *Azadirachta indica* A. Jus showed positive results for IAA acid production, phosphate solubility, and siderophore production and it was determined that these isolates had high performance in the development of tomato (Verma et al., 2011). A *Streptomyces *isolate was proven to be useful under salt stress conditions in the work of Sadeghi et al. (2018), who utilized this isolate as an enhancer in the development of wheat plants (Sadeghi et al., 2012). Secondary metabolites produced by microorganisms enable plants to resist infections and hence these metabolites increase the chance of plant survival (Sousa et al., 2008). Endophytic *Streptomyces *particularly produce biocontrol agents and bioactive natural products including fistupyrone and cytokinin-like metabolites (Verma et al., 2011). 

Plant growth promotion potential of *Streptomyces *members in different plants was investigated and results indicated an increase in plant growth through different mechanisms (Sousa et al., 2008; Sadeghi et al., 2012). Consequently, discovery of new species and metabolites of PGPR will certainly contribute to the investigations conducted in industrial, biotechnological, and agricultural areas. Therefore, the objectives of the present study are to isolate members of *Streptomyces *from *Humulus lupulus* rhizosphere and determine their potential to be used as plant growth-promoting agents with a group of tests. Selected isolates were applied in plant trials. Effects of microorganisms on plant growth promotion and on nutrient uptake were evaluated with different analyses of the samples obtained from pot trials.

## 2. Materials and methods

### 2.1. Isolation of Streptomyces spp. from rhizosphere samples

A total of seven soil samples obtained from *Humulus lupulus* (European hop) rhizosphere in Pazaryeri (Bilecik, Turkey) were utilized for isolation. Samples were air-dried at room temperature for 15 days and triturated via pestle before using them. A dilution plate technique was used in the course of study to facilitate selective isolation of *Streptomyces *spp. Samples were dried for 1 h at 100 °C prior to isolation. Isolates were incubated in selective agar media such as humic acid vitamin agar (HVA), tryptone yeast glucose extract agar (TYGA), glucose yeast malt extract agar (GYMA), and tryptic soy agar (TSA). These media included cycloheximide (50 µg mL–1), rifampicin (0.5 µg mL–1), and nalidixic acid (10 µg mL–1) as antibiotics. The ingredients of media utilized in the course of the study are summarized in Supplementary Table 1. 

**Table 1:** Tests for determining PGP potential of selected isolates.

Isolate code	N fixation	Cellulase	Caseinase	IAA production	Phosphate solubilization
HCA 03	+	-	-	-	-
HCI 12	+	-	+	+	+
HCI 13	+	-	+	-	-
HCI 32	+	-	+	+	-
HCI 50-1	+	-	+	-	-
HCI 50-2	+	+	-	+	-
HCIG 03	ND	+	+	-	-
HCIG 04	ND	+	-	-	-
HCIG 09	-	-	+	+	+
HCIG 10	ND	-	-	-	-
HCIG 14	ND	-	-	+	-
HCIG 17	+	-	-	+	-
HCIG 20 (3)	+	-	-	-	-
HCIG 26-2	+	-	+	-	-
HCIG 31	+	+	-	-	-
HCI 36	+	+	+	+	+
HCV 01		-	-	-	-

ND: Not determined.

The dilution plate technique, as the name suggests, consists of serial dilutions that are used to regulate the amount of microorganisms obtained from each suspension. One gram of each sample was initially suspended in 9 mL of sterile Ringer’s solution, which was the first 10–1 dilution. These dilutions (prepared for seven soil samples) were then serially diluted to 10–2, 10–3, and 10–4 fold by adding Ringer’s solution. Solutions of 200 µL were taken from these dilutions and inoculated on selective media. Incubation was conducted at 30 °C and 45 °C for 10–14 days (Sembiring et al., 2000). Finally, the obtained isolates were preserved in 25% glycerol (v/v) at –20 °C for use in the course of this study and for future applications.

### 2.2. DNA extraction, PCR, and sequencing 

All the samples selected for DNA isolation were incubated either in tryptone yeast glucose broth (TYGB) or glucose yeast malt extract broth (GYMEB) media at 30 °C for 3–7 days.

The genomic DNA was obtained by using a modified guanidine thiocyanate DNA isolation kit (Invitrogen). DNA samples were controlled by using agarose gel electrophoresis. 16S rRNA genes of test isolates were amplified with 27f and 1525r primers (Lane, 1991). GoTaq Hot Start Master Mix was used for polymerase chain reactions (PCRs) (Supplementary Tables 2 and 3). 

Following PCR amplification, 16S rRNA gene sequences of the products were determined by 3–5 different oligonucleotide primers in an ABI 3730XL automatic sequencing device. The sequences were obtained comparatively and manually via the MEGA 6.0 program in the presence of 27f, MG3f, MG5f, 800r, and 1525r primers (Supplementary Table 3)****(Tamura et al., 2013). Results were evaluated to determine phylogenetic relationships.

The EzTaxon-e server (https://www.ezbiocloud.net) was utilized to detect the resemblance to the closest species. 16S rRNA sequences (~1500 nucleotides) of organisms and sequences belonging to the type strain obtained from DDJM, EMBL, and NCBI GenBank were aligned in MEGA 6.0 and their phylogenetic analyses were executed. Phylogenetic trees were obtained for the neighbor-joining algorithm (Saitou and Nei, 1987) following the Jukes–Cantor method (Jukes and Cantor, 1969). The bootstrap analyses of phylogenetic trees were obtained with 1000 replicates in MEGA 6.0.

### 2.3. Plant growth-promoting assays

#### 2.3.1. Proteolytic (caseinase) activity

 Isolates were tested to determine their plant growth promotion potential. Proteolytic activity was determined according to a modified procedure by using medium consisting of nutrient agar (NA) and skim milk. The strains were spot-inoculated on this medium and incubated for 3 days at 37 °C. Proteolytic activity of strains was evaluated based on formation of a clear halo zone (Kazanas, 1968).

#### 2.3.2. Cellulase activity

Cellulase activity was determined based on a method described elsewhere (Li et al., 2018). Test isolates were inoculated on CMC agar (5 g tryptone, 5 g yeast extract, 1 g KH2PO4, 0.2 g MgSO4.7H2O, 10 g NaCl, 10 g CMC, 15 g agar per liter) medium (pH 10.0) and incubated overnight at 37 °C. Cells forming a yellow hydrolysis zone were considered as cellulase (glucanase)-positive. 

#### 2.3.3. Phosphate solubility 

The solubilization of insoluble phosphate was examined in cultures of test strains grown in the National Botanical Research Institutes phosphate-bromophenol blue (NBRIP-BPB) medium (Nautiyal, 1999). The colonies solubilizing phosphate were determined based on formation of a clear halo zone following an incubation period at 30 °C for 7 days. 

#### 2.3.4. IAA test

Test isolates were grown in LB broth supplemented with 100 mg/L of L-tryptophan for indole acetic acid determination, which was conducted in comparison with a control (LB broth). Strains were incubated in the dark at 30 °C for 7 days. At the end of incubation, cultures were centrifuged at 10,000 rpm for 15 min to obtain 1 mL of supernatant in which 2 mL of Salkowski’s reagent (2 mL FeCl3 (1.35% wt/wt), 49 mL water, and 49 mL 60% (v/v) perchloric acid)**was added. Then the resulting solution was allowed to stand for 30 min in the dark (Gordon and Weber, 1951). Pink color formation at the end of the designated time interval indicated IAA production. 

#### 2.3.5. Growth of isolates in nitrogen-free medium 

Growth of isolates in nitrogen-free medium was conducted based on the method of Trujillo et al. (2010). Isolates were inoculated inside test tubes with 10 mL of semisolid agar (1% Yeast Carbon Base and 1% Noble Agar) and the strains were incubated in the dark at 28 °C for 3 weeks. Semisolid agar supplemented with (NH4)2SO4 (2 g L–1) was used as a positive control.

### 2.4. Pot trials with tomato plants to determine PGPR potential of the strains

Two microorganisms (HCI 12 and HCI 36) were determined by evaluation of enzyme tests results and these were utilized as bioinoculants in tomato production. 

Tomato**(*Solanum lycopersicum*) seeds were sterilized with 10% sodium hypochlorite for 8 min and rinsed with sterile distilled water three times prior to experiments. Soil culture experiments were conducted in the presence of a peat/vermiculite/perlite mixture (2/1/1). A total of 50 seeds were either inoculated in 30 mL of HCI 12 and HCI 36 containing LB broth (A600 = 0.1 OD) for 2 h or treated with sterilized deionized water to be evaluated in control experiments. Following the inoculation/water treatment procedure, seeds were sown in pots with 1 seedling in each pot. The seedlings from both culture systems were maintained in a climate room at a day/night temperature of 26/23 °C with 450 μmol photons m–2 s–1 of light supplied for 16/8 h light/dark and 42% relative humidity at all times. 

Germination % was determined at the end of the 5th day, 25 tomato plants were harvested at the end of the 15th day, and 25 tomato plants were harvested at the 21st day of the experiment in order to determine their shoot lengths, root lengths, dry/fresh weights, and vigor index values. Experiments were performed as 3 separate runs with 3 replicates.

#### 2.4.1. Vigor index and biomass analysis

Vigor index (VI) was calculated according to a formula using the germination %, root length, and shoot length values obtained in the course of experiments:

Vigor index = (mean root length + mean shoot length) × germination % (Supplementary Tables 4a and 4b).

Fresh (FW) and dry (DW) weights of tomato plants were measured on the 15th and 21st days of the experiment. Total biomass of fresh samples was calculated as the sum of root and shoot weights. Samples were dried at 70 °C for 48 h prior to analyses. A similar procedure was applied in determination of total dry weights. Total fresh and dry weights of samples were presented as % change compared to the control (Supplementary Tables 5a–5d). Experiments were repeated three times. 

### 2.5. FTIR analysis

An FTIR spectrometer (PerkinElmer, USA) equipped with a universal ATR Miracle accessory was used to analyze the leaves of plants obtained from inoculated seeds and the control. The leaf sample was placed on a ZnSe crystal plate (PerkinElmer) and FTIR spectra were recorded over the region of 4000–380 cm–1 at room temperature, with a resolution of 4 cm–1 and 64 scans. First, raw data (first derivates) were smoothed using IRSolution software (Shimadzu, Japan). The second derivative and vector-normalized IR spectra were used in all data analyses while the signals of amide and lipid bands were analyzed from the vector-normalized absorbance IR spectra. Peak intensity was determined using the Quant option in the IRSolution software. Baseline corrected spectra were normalized according to the following formula: (x – min(x))/max (x – min(x)), with x as the absorbance. Principal component analysis (PCA) was performed using MATLAB software. Each experiment was repeated three times. 

### 2.6. Determination of nutrient uptake

Tomato plants obtained on the 15th and 21st days of the experiments were analyzed to determine their total nitrogen, phosphorus, and potassium amounts using spectrophotometric and atomic absorption spectrometry methods (Fontaine, 1942). 

### 2.7. Biofilm formation potential

Biofilm formation potential was determined via modification of the methods previously described in the works of Ma and Wood (2009) and Kaiser et al. (2013). Tests were conducted in petri dishes in the presence of agars containing Congo red and LBnos. 

The biofilm formation potential of strains was also determined by a well plate method with slight modifications (Winn et al., 2014). Congo red agar contained 10 g of peptone (casein), 5 g of yeast extract, 0.004% congo red, 0.002% Coomassie brilliant blue G 250, and 15 g of agar. LBnos (LB without addition of NaCl) agar included 5 g of yeast extract, 10 g of peptone (casein), and 15 g of agar. HCI 12 and HCI 36 isolates were point-inoculated on both media. Following the incubation process, biofilm formation was evaluated in terms of color change in the colonies in the case of agars containing Congo red. The strains with reddish-black color and rough colony formation were evaluated as positive for biofilm formation and pink-red colony formations of strains on Congo red agar were interpreted as negative for biofilm formation. LBnos was utilized as a medium in order to determine curly formation and/or cellulose during biofilm formation. Evaluation was conducted based on the growth of strains in this medium. 

Freshly prepared cultures were diluted with LB broth medium to OD600 0.05 and then 100 µL was transferred to LB broth medium. Biofilm formation was observed by incubation for 24 h at 160 rpm at 37 °C. After incubation, the medium was removed and the tubes were gently washed three times with distilled water. Biofilm structure was validated by staining the tubes with 1% crystal violet for 20 min. Crystal violet was then removed after washing 3 times with distilled water. Biofilm formation was evaluated as positive with the occurrence of visible film linings (pellicle) along the walls of the tube.

### 2.8. Statistical analysis

Statistical analyses were performed using SPSS for Windows, including one-way analysis of variance (ANOVA) and Tukey’s pairwise comparisons. The t-test was utilized as a hypothesis testing tool. PCA was performed using MATLAB software.

## 3. Results and discussion

### 3.1. Isolation of Streptomyces spp.

Potential *Streptomyces* spp. were isolated from *Humulus lupulus* rhizosphere by using selective HVA, TYGA, GYMA, and TSA media. The isolates that were thought to belong to different actinomycete groups were transferred to tryptone yeast glucose extract medium for purification. At the end of the incubation process, each purified isolate was stocked in 25% glycerol at –20 °C. A total of 68 isolates were obtained at the end of isolation processes. 

### 3.2. 16S rRNA sequence data results, analysis, and phylogenetic tree formation

Thirty of 68 isolates were selected based on morphological and microscopic determination. A total of 18 organisms among 30 were determined to be closely related to *Streptomycetes *spp. at the end of 16S rRNA analyses. 

**Figure 1:**
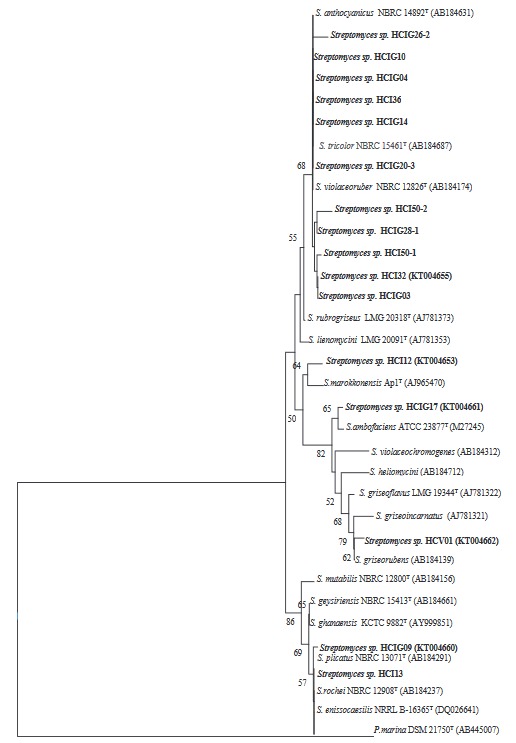
Neighbor-joining (Saito and Nei, 1987) phylogenetic tree of 16S RNA genes of test organisms and type strain of the genus Streptomyces. Nodes with 1000 replicates and bootstrap values above 50% are shown; 0.1% substitutions per nucleotide position and P. marina used as out-group.

*Streptomyces* sp. HCI 32, HCI 50-1, HCI 50-2, HCIG 03, HCIG 04, HCIG 10, HCIG 14, HCIG 20-3, HCIG 26-2, HCIG 28-1, HCIG 31, and HCI 36 isolates had 100%–98.96% similarity to the *S.* *anthocyanicus* type strain with 0–8 nt difference. *Streptomyces *sp. HCI 12 had 99.4% similarity to *S. marokkonensis* with a difference of 9 nucleotides. The HCI 13 *Streptomyces* isolate had 100% similarity with *S. rochei*. The HCIG 09 *Streptomyces* isolate had 99.93% similarity to *S. plicatus* with 1 nucleotide difference. Finally, the *Streptomyces* sp. HCIG 17 isolate had 99.46% similarity to *S*. *ambofaciens* type strain with 8 nt difference (Figure 1). 

Members of the *Streptomyces *genus, a known member of the phylum Actinobacteria, are widely found in soil and play an important role in maintaining the biological balance in soil, especially by producing substances like enzymes and antibiotics. They are also capable of establishing symbiotic relationships with plants and, thanks to all these properties, they can be used in plant development. *Streptomyces *isolates obtained from an endophytic plant were selected for use in plant trials in the present study. 

### 3.3. Plant growth promotion assays

Plant growth-promoting properties of 18 isolates were tested by measuring IAA production, phosphate solubility, cellulase activity, and caseinase activity. They were also tested for their growth. Three strains indicated positive results for phosphate solubility, 7 isolates were positive for IAA production, 8 isolates were positive for caseinase activity, and 5 isolates were positive for cellulase activity. The test isolates that indicated positive results are shown in Table 1. 

The main idea in utilization of phosphate-solubilizing bacteria (PSB) is to increase the amount of usable phosphate being supplied periodically during plant growth. The HCI 12, HCIG 09, and HCI 36 isolates were determined to have phosphate solubilization ability.

Different genera such as *Bacillus*, *Rhizobium*, and *Streptomyces* have been associated with increases in crop yields due to their phosphate solubilization ability (Rodríguez and Fraga, 1999). The use of phosphate already present in the soil by PSB would result in reduction of the amount of externally supplied chemical fertilizers. PSB would therefore enhance productivity due to elevated P uptake in their presence and enable more sustainable and ecofriendly production (Sathya et al., 2017). 

IAA produced by bacteria is proven to enhance plant growth by increasing root surface and length. Microorganisms producing IAA were also reported to have indirect effects in plant growth by inducing defense mechanisms (Venturi and Keel, 2016). The HCI 12, HCI 32, HCI 50(2), HCIG 09, HCIG 14, HCIG 17, and HCI 36 isolates were found to have IAA production ability. 

Microorganisms possessing caseinase activity enhance mineralization/immobilization of N in soil. Increase of soluble N in soil would be beneficial for both plants and microorganisms. In addition to their obvious effects on plant growth, these microorganisms would also enhance the amount and kind of microorganisms in soil rhizosphere and maintain sustainability in the ecosystem they are involved in (de Oliveira Garcia et al., 2002). Caseinase activity was also associated with the biological control potential of the strain because of the lysis of the cell walls of the fungi. The HCI 12, HCI 13, HCI 32, HCI 50 (1), HCIG 03, HCIG 09, HCIG 26 (2), and HCI 36 isolates demonstrated proteolytic activity in the course of this study. The HCI 50(2), HCIG 03, HCIG 04, HCIG 31, and HCI 36 strains were shown to secrete cellulase, which is important in cultivation in the presence of organic fertilizers. The presence of cellulase-degrading microorganisms would increase both nutrition of soil and microorganism flora, which would increase crop efficiency. 

The HCA 03, HCI 12, HCI 13, HCI 32, HCI 50(1), HCI 50(2), HCIG 17, HCIG 20 (3), HCIG 31, and HCI 36 strains indicated good growth performance in nitrogen-free semisolid medium.

Selection criteria of the isolates to be used in pot trials were determined based on the results of these tests. Evaluation of the tests revealed that HCI 12 had positive results for all tests except cellulase activity and HCI 36 was positive in all tests (Table 1). Hence, these two strains were selected for plant trials. 

### 3.4. Assessing microorganisms’ performance in the growth of tomato plant

**Figure 2:**
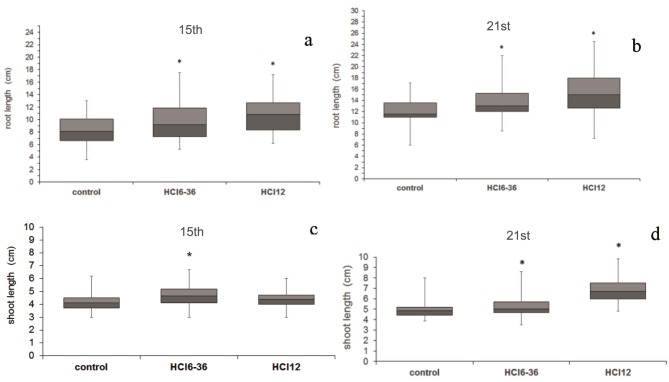
Effect of HCIG 36 and HCI 12 on a, b) root and c, d) shoot length of tomato plants grown in soil. *: Significant at P < 0.05.

**Figure 3:**
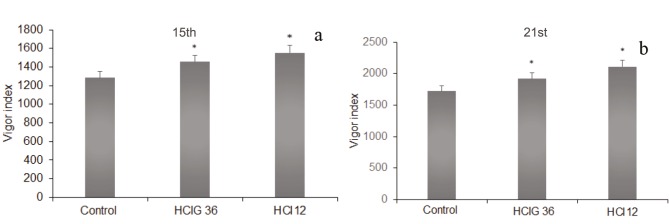
a, b) Effect of HCI 12 and HCIG 36 strains on vigor index of tomato plants grown in soil. *: Significant at P < 0.05.

The initial step in pot trials was evaluation of germination %, which was determined on the 5th day of the experiment. Although no significant difference was observed when compared to the control, preliminary results in the presence of strains were promising with 100% germination compared to 99% germination in control seeds. Twenty-five plants were harvested at the end of the 15th day. The plants inoculated with HCl 12 were determined to have longer roots than the control and those inoculated with HCI 36. The results obtained with plants harvested on the 21st day were consistent with those obtained at 15 days (Figures 2a and 2b). There was no significant difference in shoot lengths between the plant groups inoculated with microorganisms and the control group at the end of 15 days. On the other hand, when compared to the control, a significant improvement in shoot lengths was observed via HCI 12 and HCI 36 utilization after 21 days (Figures 2c and 2d). Vigor index values calculated by evaluation of germination rate and shoot and root lengths are illustrated in Figures 3a and 3b. Results obtained for both the 15th and 21st days showed higher values in the presence of HCI 12 (1.22 times) and HCI 36 (1.11 times) than with the control, as expected (Figures 3a and 3b). 

**Figure 4:**
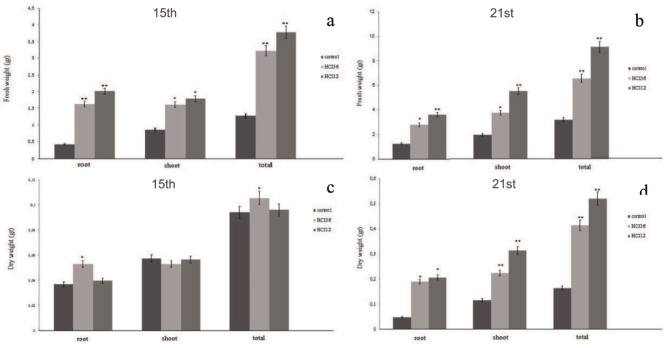
a, b) Fresh weights and c, d) dry weights of tomato plants after seed inoculation with HCI 12 and HCI 36. Control group is given for comparison. *: Significance at P < 0.05, **: P < 0.01.

Based on the results obtained so far, it could easily be concluded that the selected strains were successful in terms of improving plant growth. On the other hand, the highlight of the study is the increase in the biomass values of the plants whose seeds were inoculated with these strains. A noticeable increase was observed in root and shoot fresh and dry weights in the presence of both the HCI 12 and HCI 36 strains (Figures 4a–4d). Total fresh biomass of plants inoculated with HCI 12 and HCI 36 was much higher when compared to the control (295.31% and 251.56%, respectively) at the end of 15 days. This increase was determined as 284.42% and 204.67% in the presence of HCI 12 and HCI 36 at the end of 21 days. The increase in total dry biomass of whole plants at the end of 15 days was determined as 103.1% and 116.6% for HCI 12 and HCI 36, respectively. The increase in total dry biomass at the end of 21 days was notable with 317.07% for HCI 12 and 252.43% in the presence of HCI 36 (Figures 4a–4d). 

Numerous studies have recently been conducted on the effect of *Streptomyces* isolates on the development of different plants and as biocontrol agents (Tokala et al., 2002; Sousa et al., 2008; Verma et al., 2011; Sadegni et al., 2012; Gong et al., 2018). Endophytic *Streptomyces* strains isolated from *Azadirachta indica* A. Jus were shown to have high performance in the development of tomato. The microorganism had positive results for IAA acid production, phosphate solubility, and siderophore production (Verma et al., 2011). The germination rate and highest root length were determined as 81%–84% and 7.61 cm, respectively. This study had high similarity to our study based on the investigated plant and species. *Streptomyces *isolates had similar effects on plant roots and shoots, whereas isolates utilized in this study were determined to have an effect particularly on the roots of the plant. 

*Streptomyces* isolates have been successful in a variety of plants such as *Pisum sativum *and in* Acromyrmex octospinosus *(Tokala et al., 2002; Seipke et al., 2011). *Streptomyces *strains were proven to be useful in stress conditions in the work of Gong et al. (2018), who utilized 2 isolates as an enhancer in the development of wheat plant. Results indicated higher plant growth under salt stress conditions (Gong et al., 2018). Although there are many publications about the use of rhizosphere-derived isolates in plant growth promotion of different plants (Vasconcellos et al., 2010), a plant growth-promoting study with this type of strain has not been found in the literature and an important contribution to the existing *Streptomycetes* spp. was provided with this study.

### 3.5. FTIR analysis

**Figure 5:**
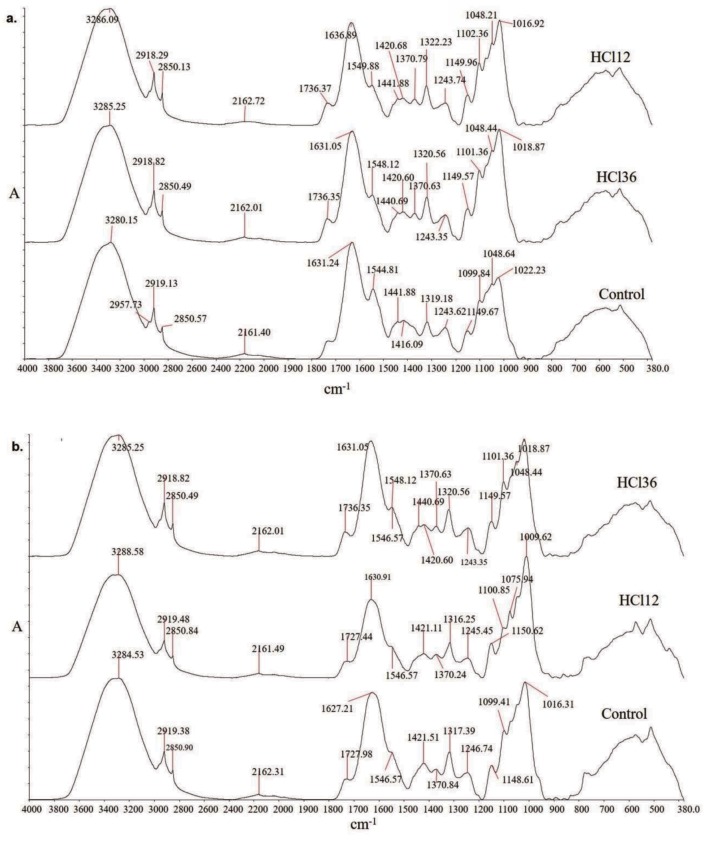
FTIR spectra of tomato leaves inoculated with HCI 12 and HCI 36 and noninoculated (as control): a) 15th day, b) 21st day.

The FTIR spectra of tomato leaves inoculated with HCI 12 and HCI 36 along with noninoculated (control) samples are illustrated in Figures 5a and 5b (a: 15th day; b: 21st day). Previous studies demonstrated that FTIR spectra of proteins and total lipids were present in the region of 3000–2800 cm–1 (Kothari et al., 2013). In this study, the bands observed at 1737.9 cm–1 were noted to be the characteristic bands of fatty acids due to the C=O mode of the side chain from the ester carbonyl group (Dean et al., 2010). Previous FTIR studies also reported that the spectral region between 1200 and 950 cm–1 was identified as having the characteristic bands for C-O, C-C, C-O-C, and C-O-P stretching of polysaccharides. Brandenburg and Seydel (1996) also showed that the v(C-O-C) stretching of the polysaccharides region was observed at 1050 and 1012 cm–1 (Brandenburg et al., 1999). Based on this knowledge, the bands observed at 1074, 1047, and 1012 cm–1 were evaluated as the validation of the presence of polysaccharides in tomato leaf (Figures 5a and 5b). The characteristic bands observed at 1654 cm–1 were identified as amide I. 

The changes in amide, carbohydrate, and lipid structures were investigated with PCA conducted in selected regions. Three regions of the absorption spectrum characteristic for amide, carbohydrate, and lipid were determined as 1700–1500 cm–1, 1500–1200 cm–1, and 3000–2800 cm–1, respectively (Kothari et al., 2013; Cabra Cendales et al., 2017; Bortoletto et al., 2018; Bouyanfif et al., 2019). The PCA results clearly demonstrated changes in protein, carbohydrate, and lipid fingerprint regions compared to the control (Supplementary Figures 1–3). 

PCA was conducted for samples obtained at the 15th and 21st days of the experiment. These values were evaluated in order to determine the change due to the response of microorganisms. PC-1 and PC-2 % values were calculated for protein, carbohydrate, and lipid fingerprint regions of samples obtained at the end of the 15th and 21st days. PC-3 analyses were not conducted as results indicated the change of the two major compounds with total variance of 100%. In the PCA of the amide region, there was a change in the variance of PC-1 and PC-2 between day 15 and 21 samples, which might have been related to microorganism inoculation. Similar changes were observed for lipid and carbohydrate structures (see Supplementary Table 6). The variance obtained for PCA was particularly high for carbohydrates. Considering the quantitative changes obtained by FTIR intensities, results show the implication of an alteration in the structure of carbohydrate and lipid metabolisms.

In the present study, FTIR results were also evaluated to provide a comparison of peak intensity ratios of carbohydrate/amide at the end of the 15th day. Results indicated 1.4-fold higher values in the presence of HCI 12 compared to HCI 36. This was an expected result considering the higher effect of HCI 12 on carbohydrate and amide structures when compared to the control. The mean ratio of the peak intensity of lipid/amide I increased approximately 2-fold in the inoculation process conducted with HCI 12 and HCI 36 when compared to control groups, which indicated an enhancing effect of both microorganisms on lipid biosynthesis.

The preliminary study of FTIR and PCA helped us to determine amide, carbohydrate, and lipid exchange in microorganism-inoculated plants compared to the control.

### 3.6. Determination of nutrient uptake

Samples obtained from tomato at the end of the 15th and 21st days were analyzed in order to determine the effect of 2 h of microorganism inoculation on nutrient uptake. Results obtained from samples at 15 days indicated a significant increase of K uptake in the presence of HCI 36 and HCI 12 compared to the control. K values varied as HCI 36 > HCI 12 > control for 15-day samples. However, HCI 12 was found to be more effective than HCI 36 and the control at the end of the 21st day. 

**Figure 6:**
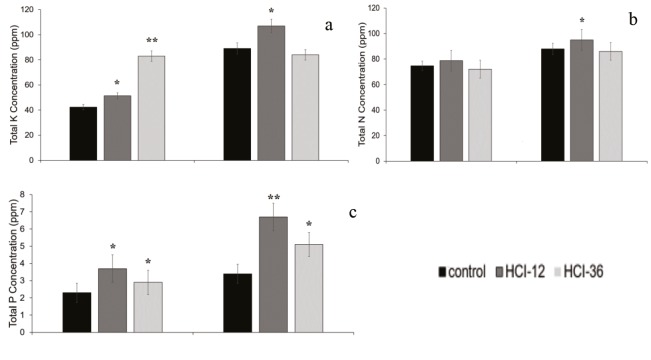
Effect of HCI 12 and HCI 36 strains on nutrient uptake of tomato: a) K, b) N, and c) P. *: Significance at P < 0.05, **: P < 0.01.

The performance of HCI 12 was also higher in terms of P uptake regardless of sampling time. P uptake varied as HCI 12 > HCI 36 > control. The presence of HCI 12 and HCI 36 strains increased the P uptake in tomato at the end of 15 and 21 days. The performance of HCI 12 was particularly higher than that of HCI 36 at the end of 21 days. Besides K and P, N amounts obtained in the presence of HCI 12 were also higher than those with HCI 36 at the end of 21 days. As a conclusion, based on the results, it can be said that HCI 12 was the most effective microorganism utilized in enhancement of plant growth (Figure 6). 

### 3.7. Biofilm formation potential

Biofilms are common life habitats that are formed in the presence of moist environments, which are formed as embedded in polysaccharides secreted by bacteria (Rodriguez-Navarro et al., 2007). The symbiotic areas of roots and microorganisms, which are called rhizosphere, are suitable for biofilm formation due to sufficient moisture. Studies indicated their direct effect on root colonization (Dietel et al., 2013). Biofilm formation also facilitates the diffusion of nutrients and metabolic compounds and plays an important role in plant survival in competitive environments (Noirot-Gros et al., 2018).

**Figure 7:**
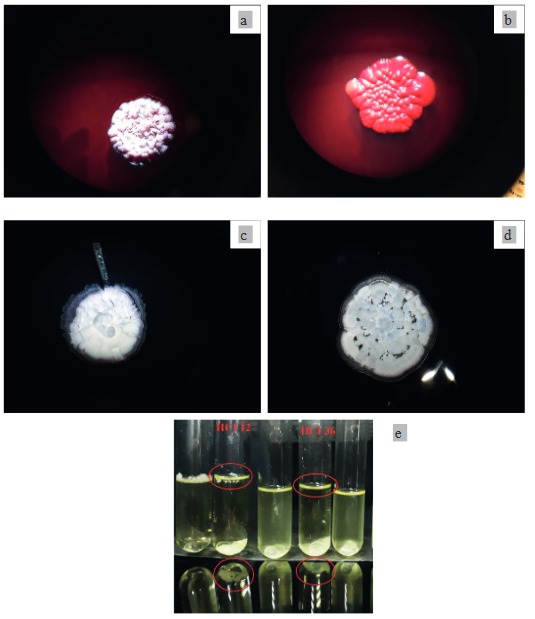
a) Growth of HCI 12 isolate in Congo red, b) growth of HCI 36 isolate in Congo red, c) growth of HCI 12 in LBnos agar, d) growth of HCI 36 in LBnos agar, e) modified well plate method results of HCI 12 and HCI 36 isolates.

The HCI 12 and HCI 36 strains utilized in the present study were evaluated as positive for biofilm formation potential due to the observation of reddish colony formation of strains following their inoculation on Congo red (Figures 7a and 7b). However, it was difficult to conclude that HCI 12 was positive due to the spore formation of this isolate. Results of these isolates on Congo red were also confirmed by LBnos medium and the modified well plate method. Growth of both strains on LBnos medium was considered as a positive result (Figures 7c and 7d). The modified well plate method also indicated positive results with the occurrence of pellicle along the walls of the tubes. Pellicle formations for HCI 12 and HCI 36 are given in Figure 7e. 

### 3.8. Conclusions

Sixty-eight isolates were obtained via application of procedures specific to screening of actinomycetes. 16S rRNA analysis revealed the presence of *Streptomyces* species with 99.4% similarity and 9 nt difference in the case of HCI 12 and 99.4% similarity and 8 nt difference in the case of HCIG 17. These strains were determined as potential novel species, which was one of the highlights of the present study. The accession numbers of these potential novel species are given in Figure 1 and Supplementary****Table 7. 

The plant growth-promoting properties of the selected 18 isolates were screened and plant growth-promoting effects of two isolates were investigated for tomato plants based on the test results. Plant trials conducted in the presence of HCI 12 and HCI 36 revealed significant increases in fresh weight root length and vigor index values. The increase in these values along with enhanced nutrient uptake in their presence was attributed to their IAA production ability, phosphate solubilization, nitrogen fixation ability, and biofilm formation potential. Results indicated higher N, P, and K uptake values for HCI 12 and HCI 36 when they were compared to the control group. Microorganism inoculation also affected carbohydrate, amide, and lipid production in tomato as shown via FTIR analyses. A significant increase was observed in carbohydrate/amide and lipid/amide I ratios of plants inoculated with HCI 12 and HCI 36. The changes in carbohydrate, amide, and lipid metabolisms were validated via PCA. Consequently, results indicated two microorganisms as potential candidates for use as PGPR and also showed an important contribution to existing *Streptomyces* utilized as PGPR.

## Acknowledgments

This research was supported by Bilecik Şeyh Edebali University, Project No. 2013-01.BİL.13-01 and the valuable contributions of Assoc. Prof. Dr. Dilek Ünal, Assist. Prof. Y. Emre Şimşek, and Assoc. Prof. Dr. Levent Değirmenci are gratefully acknowledged. 
